# Pathopsychological assessment of pediatric patients with autoimmune diseases of central nervous system

**DOI:** 10.1192/j.eurpsy.2021.1676

**Published:** 2021-08-13

**Authors:** J. Fedorova, N. Burlakova, J. Mikadze, U. Azizova

**Affiliations:** 1 Department Of Neuro- And Pathopsychology, Lomonosov Moscow State University, Moscow, Russian Federation; 2 Department Number 1, Russian Children’s Clinical Hospital, Moscow, Russian Federation

**Keywords:** CNS disease, autoimmune disease, pathopsychological assessment

## Abstract

**Introduction:**

Autoimmune diseases of central nervous system (CNS) are wide spread in children. In some cases, mental disturbances in such patients are barely noticeable in the beginning, which hinders early detection of risks in the child’s mental development.

**Objectives:**

The study focuses on comparative analysis of the structure of mental disorders in pediatric patients with autoimmune diseases of CNS.

**Methods:**

Research includes two cases: girls aged 14 and 16, one with acute disseminated encephalomyelitis (ADEM), disease onset at 4 years and 11 months, and another with multiple sclerosis (MS), disease onset at 5 years and 5 months. The following methods were used: analysis of patient’s medical record, interview with neurologists, pathopsychological assessment.

**Results:**

Common features in both cases: 1) organic brain disorders; 2) patients do not demonstrate intellectual deterioration, can master regular school curriculum; 3) detected mental disturbances reflect risks for mental and personality development. Specific features: 1) the patient with MS demonstrates polymorphism of mental disorders, while the patient with ADEM — homogeneity of mental disorders; 2) main problems of the patient with MS are related to self-regulation, which makes the general picture similar to pseudo-frontal syndrome; the patient with ADEM has major neurodynamic disturbances, which has similarity to psychoorganic syndrome; 3) predictors of personality disorders detected in case of MS determine the negative prognosis for mental development.

**Conclusions:**

The delineated features evidence for further psychological study of CNS autoimmune diseases and formulation of criteria for clinical psychological assessment. These patients need to be monitored by psychologists to prevent personality disorders.

**Disclosure:**

No significant relationships.

